# Dectin-1 and TIM3 Expression in Deep Vein Thrombosis of Lower Limbs (DVTLL)

**DOI:** 10.3390/jcm9113466

**Published:** 2020-10-28

**Authors:** Vincenza Barresi, Salvatore Napoli, Giorgia Spampinato, Daniele Filippo Condorelli, Salvatore Santo Signorelli

**Affiliations:** 1 Department of Biomedical and Biotechnological Sciences, Section of Medical Biochemistry, University of Catania, 95123 Catania, Italy; barregi@unict.it (V.B.); napo.salvo@gmail.com (S.N.); giorgiaspampinato@unict.it (G.S.); daniele.condorelli@unict.it (D.F.C.); 2 Department of Clinical and Experimental Medicine, University of Catania, 95123 Catania, Italy

**Keywords:** deep vein thrombosis, inflammation, immunity, T lymphocyte, Dectin-1, TIM3

## Abstract

The pathophysiological mechanisms of venous thromboembolism are venous stasis, endothelial damage, and hypercoagulability, while less attention has been given to the role of both innate and native immunity. In this paper, we investigate the involvement of the activated immune system detected through some indicators such as TIM3 and Dectin-1 expressed by T lymphocytes. TIM3 and Dectin-1, two surface molecules that regulate the fine-tuning of innate and adaptive immune responses, were evaluated in patients affected by deep vein thrombosis of lower limbs (DVTLL). CD3^+^, CD4^+^ and CD8^+^ T lymphocytes obtained from patients affected by DVTLL were analysed using fluorescence-conjugated antibodies for TIM3 and Dectin-1 by an imaging flow cytometer. DVTLL patients showed a higher number of CD4^+^ and CD8^+^ T lymphocytes. TIM3 expression in T lymphocytes was very low in both DVTLL patients and controls. On the contrary, an increase in Dectin-1^+^ cells among CD4^+^ and CD8^+^ T lymphocytes from DVTLL patients was observed. Dectin-1 is known to play a role in inflammation and immunity and our result suggests its potential involvement in thrombotic venous disease.

## 1. Introduction

There is a growing interest in venous thromboembolism (VTE) because it is a potential and serious consequence in patients affected by solid or haematologic cancer hospitalised for surgical or orthopaedic interventions, or who suffer from chronic or acute medical diseases such as heart failure, chronic obstructive pulmonary disease (COPD) [1,2] and asthma [3,4]. VTE is now ranked as the third most common cause of disability and cardiovascular death worldwide [5,6]. Endothelium dysfunction, blood stasis and hypercoagulability, which are included in Virchow’s triad, are still considered hardpoints in the pathophysiology of VTE. The aforementioned triad activates the coagulative cascade, by disrupting the fibrin structure, favouring blood cell (leucocyte, platelet) adhesion and promoting cell-derived microvesicles [7]. A growing body of evidence has shown the role played by inflammation in the initiation of a venous thrombus, the renewal of tissue integrity and function of venous circulation [6,8]. Although the role of inflammation has been analysed and the presence of T cells in the thrombus has been demonstrated [9], current guidelines for the treatment of VTE suggest using anticoagulants as the first line of treatment in VTE. To date, few contributions have been made concerning immunity and immune cells in venous thromboembolism. Recent reports in the literature show the involvement of two receptors, Dectin-1 [10] and TIM3 [11] in the immunity and inflammation conditions. Dectin-1 is expressed primarily by cells of myeloid lineage such as monocytes, macrophages, dendritic cells (DCs) and neutrophils. Dectin-1 acts as an antigen uptake receptor and triggers multiple signalling pathways. leading to NF-κB, type I interferon (IFN) and/or inflammasome activation [11]. T cell immunoglobulin and mucin domain (Tim) molecules are type 1 transmembrane proteins expressed on various immune cells and are similar to PD-1/PD-L1/2 negative regulators of immune response. They include three human members (Tim-1, -3, -4) expressed in a variety of immune cells, such as natural killer (NK) cells, monocytes, macrophages and mast cells. During the innate immune response, Tim-3 promotes inflammation via tumour necrosis factor-α (TNF-α) secretion by monocytes and antigen-presenting cells and enhances macrophage clearance of intracellular pathogens [12]. In this paper, we analyse the expression of these two transmembrane proteins in T cells from deep vein thrombosis of lower limbs (DVTLL) patients and control subjects.

## 2. Experimental Section

### 2.1. Patient Recruitment

Nine patients who were hospitalised for DVTLL in the General Medicine Unit of the University Hospital “Policlinico Rodolico” of Catania, Italy, were considered for the study. An equal number of healthy individuals, admitted to the non-invasive vascular medicine lab of our unit, was also enrolled. The patients’ characteristics are reported in Table 1. DVTLL was diagnosed by ultrasonographic examination with an ultrasound system (US) equipped with a 4 Mhz linear phased array probe (MyLaB 70, MyLab X vision, ESAOTE Italia Via di Caciolle, 15,50127 Firenze, Italy). The diagnosis criteria for DVTLL were based on an echogenic image of one of the deep veins of the lower limbs and incompressibility of the vein under US pressure (CUS positive test). When patients affected by DVTLL were enrolled for the study, they were treated with anti-thrombotic drugs (low-molecular-weight heparin) for one day. No anti-inflammatory drugs were administered to the patients. Patients were informed about the aim of study and were asked to give verbal informed consent to participate in the study and to provide blood samples. All subjects gave their informed consent before their inclusion in the study. The study was conducted in accordance with the Declaration of Helsinki, and the protocol was approved by the Ethics Committee of the Garibaldi Hospital (Catania, Italy; resolution n.23/2016/CECT2).

### 2.2. Antibodies and Fluorophores

The following five monoclonal primary antibodies directly conjugated with fluorophores according to the instrumental settings and available lasers were used:

- **anti-human CD3** antibody (clone UCHT1) conjugated with allophycocyanin (**APC**), a fluorophore with an excitation peak at 652 nm and an emission peak at 657.5 nm (Biolegend, San Diego, CA, USA cat 300439);
- **anti-human CD4** (clone OKT4) and **anti-human CD8** (SK1) fluorescein isothiocyanate (**FITC**), with an excitation peak at 495 nm and emission peak at 521 nm (Biolegend, San Diego, CA, USA cat 317408, 344704);
- **anti-Dectin-1** (14E2) and **anti-TIM3** (clone F38-2E2) were conjugated with phycoerythrin (**PE**) with an excitation peak at 565 nm and an emission peak at 573 nm (Biolegend, San Diego, CA, USA cat 345006, 355404).

### 2.3. Cell Cultures for Antibody Validation

Anti-human TIM3 and anti-human Dectin-1 antibodies were validated on two immortalised cell lines of leukaemia: HL60 and K562. Cell line HL60 (ATCC, CCL-240) was obtained from the peripheral blood of a patient affected by acute promyelocytic leukaemia, with myeloblastic morphology. K562 cells (ATCC, CCL-243) were obtained from the bone marrow of patients with chronic myelogenous leukaemia, with lymphoblastic morphology. Single staining was performed using anti-TIM3-PE and anti-Dectin-1-PE antibodies, according to the Biolegend protocol [www.biolegend.com]. Cells were cultured at high confluence in RPMI 1640 (w/HEPES w/Glutamax-I, cat 72400021, Gibco, Thermo Fisher Scientific) supplemented with 10% fetal bovine serum (FBS), and 1% penicillin/streptomycin. The cell cultures were incubated at 37 °C in a humidified atmosphere with 5% CO_2_ and 95% air. The medium was changed twice a week. Cells were counted and resuspended at 1,000,000 cells/mL and labelling with the antibodies was performed according to the Biolegend protocol.

### 2.4. Sample Preparation

In the early morning, fasting whole blood samples were collected using a Vacueteiner EDTA tube (Becton Dickinson, 1575 Airport Rd, Sumter, SC 29153, Stati Uniti USA), and immediately processed and analysed. White blood cells were isolated using the Biolegend protocol (https://www.biolegend.com, 8999 BioLegend Way, San Diego, CA 92121). The tube containing the blood was inverted several times to mix the contents. Briefly, the red blood cells were lysed using RBC lysis buffer followed by three washes with isotonic saline solution. Then, staining with the previously described five monoclonal fluorescent dye-coupled anti-human antibodies was conducted. For each patient, four aliquots of lymphocyte suspensions (100 µL) were stained with antibodies using four different combinations of antibodies (10 µL of each antibody): CD-3 + CD-4 + TIM-3 (A); CD-3 + CD-4 + Dectin-1 (B); CD-3 + CD-8 + TIM-3 (C); CD-3 + CD-8 + Dectin-1 (D). The mixture was kept in the dark, at 4 °C, for half an hour. A wash step was performed to eliminate any antibody excess, and finally resuspended in cell staining buffer (500 µL). Stained cells were analysed by cytofluorimetry. The analysis was conducted immediately to prevent signal loss due to fluorescent dye degradation. After staining, the cellular suspension was analysed on a flow cytometer (Amnis FlowSight Millipore, Merck KgaA, Darmstadt, Germany), and the results were analysed using Image Data Exploration and Analysis (IDEAS) software (Amnis part of EMD Millipore, Seattle, WA, USA) as previously reported [13] and as described in detail in the next paragraph.

**Figure 1:**
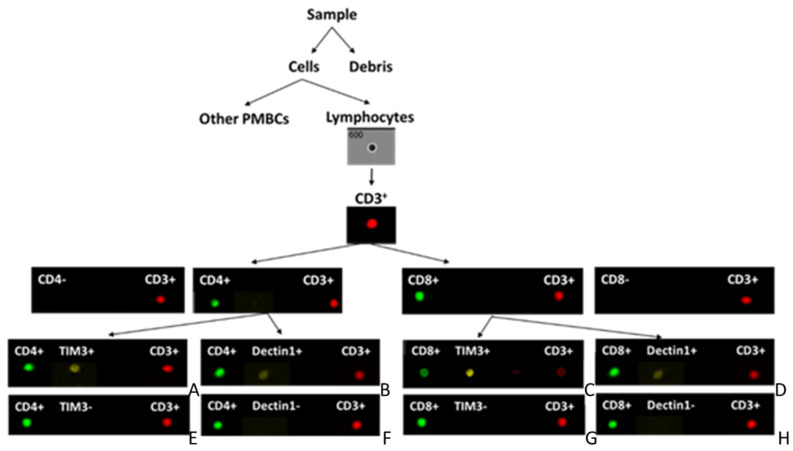
Flow cytofluorimetric analysis workflow. The image shows the sequential procedure and the microphotographs of stained cells (**A**–**H**) using five different monoclonal fluorescent dye-coupled anti-human antibodies and analysed by flow cytometry (Amnis FlowSight). For each patient, four aliquots of lymphocyte suspensions (100 µL) were stained with antibodies using four different combinations (10 µL of each antibody). Representative stained cells (event) for each of the four combinations are shown: CD-3^+^ CD-4^+^ TIM-3^+^ (**A**); CD-3^+^ CD-4^+^ Dectin-1^+^ (**B**); CD-3^+^ CD-8^+^ TIM-3^+^ (**C**); CD-3^+^ CD-8^+^ Dectin-1^+^ (**D**). Tim3^−^ and Dectin-1^−^ cells are also shown: CD3^+^ CD-4^+^ TIM-3^−^ (**E**); CD-3^+^ CD-4^+^ Dectin-1^−^ (**F**); CD-3^+^ CD-8^+^ Tim3^−^ (**G**); CD-3^+^ CD-8^+^ Dectin-1^−^ (**H**).

The algorithms in the IDEAS software create two types of functions: mask and feature. Mask refers to the set of pixels that contain the region of interest, and feature refers to calculated values using the pixel intensities of the image. Our analysis started by distinguishing the single cells from debris and cell aggregates by creating a scatter plot of “Area” versus “Aspect ratio”. The feature “Area” calculates the square microns of a given mask, while “Aspect ratio” is the ratio of the minor axis and major axis and describes how round or long an object is. The “FlowSight” is also equipped with a dedicated laser for side scattering at 785 nm, a useful tool to obtain information about cellular morphology. Therefore, creating a “single cell” gate, a scatter plot graph of “Side scatter intensity” vs. “Area”, it was possible to identify, based on morphology and area, the human peripheral blood mononuclear cells (PBMCs). Since the anti-human CD3 antibody conjugated with allophycocyanin (APC) binds the CD3 antigen expressed on the membrane surface of T lymphocytes (Figure 1), a scatter plot of “Side scatter intensity” vs. “APC intensity” was generated in order to isolate the lymphocytes from other PMBCs. The CD4 and CD8 antibodies, both conjugated with FITC, distinguished, in the scatter plot graphs of “APC intensity” vs. “FITC intensity” created based on the lymphocyte gate, two different types of lymphocyte populations: CD3^+^ and CD4^+^ cells (T helper cells) or CD3^+^ and CD8^+^ positive cells (suppressor T cells). For each graph, it was possible to recognise two types of populations CD3^+^/CD4^+^ (or CD3^+^/CD4^−^) and CD3^+^/CD8^+^ (or CD3^+^/CD8^−^). On the double-positive gates (CD3^+^/CD4^+^ or CD3^+^/CD8^+^) we finally created the histogram “Phycoerythrin (PE) intensity” to analyse the percentage of positive cells for Dectin-1 or TIM3 (both corresponding antibodies being conjugated with PE).

### 2.5. Statistical Analysis

**Figure 2:**
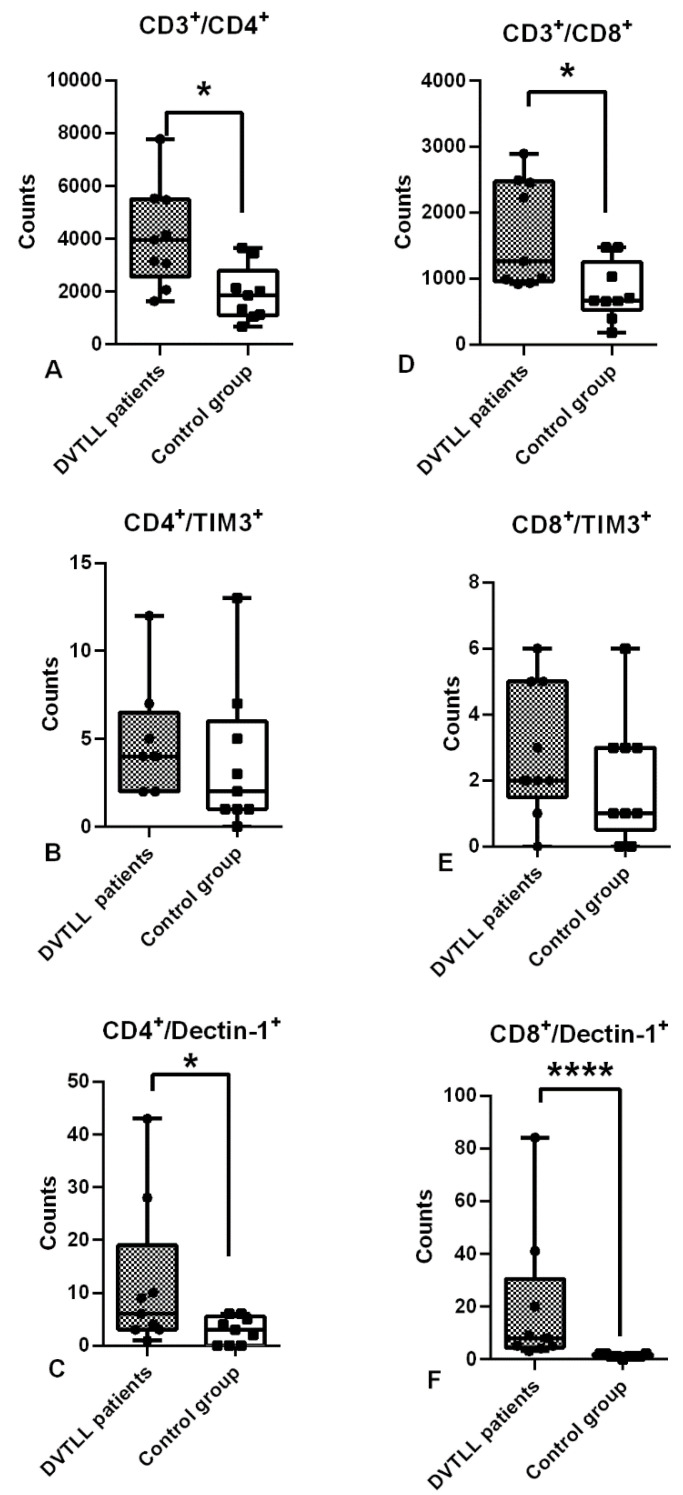
Expression of TIM3 and Dectin-1 in CD3^+^, CD4^+^ and CD8^+^ T lymphocytes belonging to DVTLL patients and healthy controls was analysed by flow cytofluorimetric analysis. The number of CD3^+^/CD4^+^ and CD3^+^/CD8^+^ cells was higher in DVTLL patients (**A**,**D**). No differences were observed for CD4^+^/TIM3^+^ and CD8^+^/TIM3^+^ cells between DVTLL patients and controls (**B**,**E**). The number of CD4^+^ and CD8^+^ cells expressing Dectin-1 (**C**,**F**) was significantly higher in DVTLL patients. Statistical analysis was performed by a Mann–Whitney test. * *p* < 0.05; **** *p* < 0.0001.

Statistical analysis was carried out using GraphPad Prism 6 (Graphpad Software Inc., La Jolla, CA, USA). A parametric unpaired *t*-test was used for the data reported in Table 1. A non-parametric *t*-test for unpaired data (Mann–Whitney U test) was performed on the data from DVTLL patients and the control group (Figure 2 and Table 2). Significance was set at *p* < 0.05.

## 3. Results

K562 and HL60 cell lines were analysed to validate the expression of TIM3 and Dectin-1 in human haematopoietic cells. K562 is a highly undifferentiated human erythroleukaemia cell population belonging to chronic myelogenous leukaemia (CML). K-562 blasts are multipotential, haematopoietic malignant cells that spontaneously differentiate into recognisable progenitors of erythrocytic, granulocytic and monocytic series. A cytofluorimeter assay showed high median values of 78.0 and 75.5 for TIM3 and Dectin-1, respectively (Table 3). HL60 is a cell line derived from a 36-year-old woman affected by acute promyelocytic leukaemia and the cells predominantly have a neutrophilic promyelocytic morphology. The cytofluorimeter assay in the last cell line also showed an equal distribution of both surface molecules, even if the median values were lower: 12.4 and 12.1 were positive for TIM3 and Dectin-1, respectively (Table 3).

The analysis performed on blood cells purified from DVTLL patients and control subjects revealed that the total count of CD3^+^ T lymphocytes isolated from peripheral blood was higher in DVTLL patients than the healthy control group, the increase was two-fold as shown by the median values (4473.3 vs. 1996.5, *p*-value < 0.0001) (Table 2). Furthermore, CD4^+^ T helper cells and cytotoxic CD8^+^ T cells were counted. The analysis demonstrated that, also in this case, both T cell populations showed higher values in DVTLL patients (mean, median, SD are shown in Table 2), two times more than in the healthy control group (CD3^+^/CD4^+^ *p*-value = 0.01; CD3^+^/CD8^+^ *p*-value = 0.018, Table 2, Figure 2A,D). Subsequently, the expression of TIM3 and Dectin-1 on the surfaces of CD4^+^ and CD8^+^ cells was evaluated. As expected, the number of TIM3^+^ on CD4^+^ and CD8^+^ cells was low. TIM3 levels did not show significant differences in expression on T helper cells (CD4^+^) and cytotoxic T cells (CD8^+^) of DVTLL patients (Figure 2B,E). On the contrary, a significant increase in CD4^+^/Dectin-1^+^ cells (*p*-value 0.027, Table 2; Figure 2C) and CD8^+^/Dectin-1^+^ cells (*p*-value < 0.0001, Table 2, Figure 2F) was observed in DVTLL patients compared to healthy controls.

## 4. Discussion

This is the first paper focusing on Dectin-1 and TIM3 measured in peripheral blood cells in DVTLL patients using the cytofluorimetric assay technique and an instrument that combines a traditional flow cytometer with a powerful and highly sensitive microscopy (FlowSight imaging flow cytometer, Amnis, Merck Millipore, Burlington, MA, USA). The three following lasers were used: 488 nm (blue), 561 nm (green) and 642 nm (red) wavelengths. Moreover, two channels were used for the brightfield. A single cell was placed in the flow chamber and when it passed through the detector of the instrument, the brightfield image of the cell and the emitted fluorescence were recorded. Another single channel was used to reveal the side-scattering (SSC) signal, which, together with the signal of the brightfield, gave information on cell morphology. The interesting feature of this instrument is the possibility to select a region on the dot-plot graph and to watch, on each channel, the relative signal of expression of proteins localised in the cell membrane, nucleus and cytosol. Figure 1 shows the workflow and the images of single cells detected for each of the single antibodies used in the analysis.

In this study, we investigated Dectin-1 [10] and TIM3 [11] expression in T lymphocytes in DVTLL patients. Indeed, Dectin-1 and TIM3 are known to play a role in several pathological conditions including infection, regulation of inflammation, allergy, transplantation tolerance, cancer, cardiovascular disease and autoimmune diseases. Our study revealed a significantly increased number of T helper CD4^+^ and T cytotoxic CD8^+^ cells in DVTLL, as well as an increased number of CD4^+^ and CD8^+^ cells expressing Dectin-1. We found a similar trend for TIM3 expression in both cellular subtypes, although the increase did not reach statistical significance.

It is well known that Dectin-1 is a receptor belonging to “C-type lectin domain family 7 member A” encoded by the CLEC7A gene (Cr12p13.2). This receptor recognises the β-glucans found in the cell walls of plants and fungi, including *Candida albicans*. Dectin-1 activation promotes both “T helper type 1” (Th-1) and “T helper type 17” (Th-17) cells in the production of interleukin (IL)-17. IL-17 is involved in cellular innate immune responses by dendritic cells that are needed in protection against fungi [14,15,16,17]. The Dectin-1 receptor is also recognised by endogenous ligands including vimentin [18], galactosylated immunoglobulins [19], and galectins [20]. Dectin-1 is highly expressed in monocytes, macrophages and neutrophils and, conversely, it is expressed to a lesser extent in B cells, T cells and dendritic cells [21], and Dectin-1 activation can influence the development of CD4 and CD8 T cells. High expression and activation of Dectin-1 causes the release of pro-inflammatory molecules [22]. Activation of the Dectin-1 receptor through spleen tyrosine kinase (SYK) protein, caspase recruitment domain 9 (CARD9) protein, adaptor proteins Bcl-10, MALT1 and complex ERK-TNK-P38 induces both NF-κB activation and the transcription of various inflammatory cells including IL-12 and TNF-α in T helper cells [18,23,24]. Pro-inflammatory cytokine IL-12 stimulates the differentiation of CD4^+^ lymphocytes into Th-1 lymphocytes; on the other hand, TIM3 has its expression restricted to inflammatory IFN-γ producing “type 1” CD4 T cells and CD8 T cells [11]. Th-1 lymphocytes are capable of activating macrophagic activity, thus they are capable of starting the phagocytosis of pathogens, and then activating other forms of defence. Furthermore, the IL-12 upregulation potentiated the cytotoxic activity of CD-8^+^ macrophages (CD-3^−^/CD-4^−^) [25]. Luther et al. 2016 [9] demonstrated that helper CD-4^+^ and cytotoxic CD-8^+^ T cells are able to infiltrate the thrombus during and after a DVT event. Effector memory T cells (T_EM_) continue to produce INF-γ in an antigen-independent manner in order to support aseptic inflammation events, such as those revealed in the DVT condition. The activation of T_EM_ cells in the thrombus leads to the recruitment of neutrophils and monocytes, delaying thrombus resolution [9]. The higher expression of Dectin-1 observed in both CD4^+^ and CD8^+^ cells suggests the involvement of Dectin-1 and its effects on Th1/Th17 cells in DVTLL. In conclusion, we suggest that there is a link between the latent chronic inflammation characterising thromboembolism and the immune system and that the role of both native and adaptive immunity should be further investigated in venous thromboembolism.

## Figures and Tables

**Table 1:** Characteristics of deep vein thrombosis of lower limbs (DVTLL) patients and control subjects (Controls).

	DVTLL Patients (*n* = 9)	Controls (*n* = 9)	*p*-Value Unpaired *t*-Test
Age (mean ± SD)	65.44 ± 18.12	58.33 ± 14.61	0.285
Weight Kg (mean ± SD)	85.67 ± 18.15	74.00 ± 19.58	0.28
Height cm (mean ± SD)	176 ± 9	173 + 5.5	0.33
Body Mass Index—BMI (mean ± SD)	27.51 + 5.11	24.86 + 6.88	0.17
Smoker (n.)	3	NA	NA

**Table 2:** Total cell counts for each stained subpopulation and percentages of stained CD3^+^ subpopulations calculated with respect to each category in DVTLL patients and in health controls are reported as mean and median. Statistical analysis for DVTLL patients compared to controls was performed by a Mann–Whitney test. Statistically significant values (*p* < 0.05) are underlined.

	DVTLL (*n* = 9)			Controls (*n* = 9)			
	Mean	Median	±SD	Mean	Median	±SD	*p*-Value (Mann–Whitney *t*-Test)
**T cell lymphocyte count (CD3^+^)**	**5283.9**	**4473.3**	**1680.8**	**1936.6**	**1996.5**	**573.4**	**<0.0001**
T Lymphocytes (CD3^+^) (as % of PBMC)	19.0	15.6	6.2	15.2	15.5	3.1	0.39
**CD3^+^/CD4^+^ count**	**4089.6**	**3962.0**	**1926.2**	**1924.7**	**1854.5**	**1045.5**	**0.01**
CD3^+^/CD4^+^ (as % of CD3^+^)	63.7	65.3	10.1	61.9	61.3	11.3	0.62
**CD3^+^/CD8^+^ count**	**1686.2**	**1263.5**	**813.2**	**805.3**	**667.5**	**441.9**	**0.018**
CD3^+^/CD8^+^ as% of CD3^+^)	27.8	26.4	6.7	28.4	29.3	9.7	>0.99
**CD4^+^/TIM3^+^ count**	**4.75**	**4.0**	**3.41**	**3.7**	**2.0**	**4.2**	**0.27**
CD4^+^/TIM3^+^ (as % of CD3^+^/CD4^+^)	0.1	0.1	0.4	0.2	0.1	0.2	0.89
**CD8^+^/TIM3^+^ count**	**2.9**	**2.0**	**2.0**	**2.0**	**1.0**	**1.9**	**0.41**
CD8^+^/TIM3^+^ (as % of CD3^+^/CD8^+^)	0.3	0.2	0.2	0.3	0.2	0.3	0.98
**CD4^+^/Dectin-1^+^ count**	**11.9**	**6.0**	**14.2**	**2.9**	**3.0**	**2.5**	**0.027**
CD4^+^/Dectin-1^+^ (as % of CD3^+^/CD4^+^)	0.3	0.1	0.4	0.2	0.1	0.2	0.65
**CD8^+^/Dectin-1^+^ count**	**19.9**	**8.0**	**26.9**	**1.2**	**1.0**	**0.7**	**<0.0001**
CD8^+^/Dectin-1^+^ (as % of CD3^+^/CD8^+^)	1.0	0.7	1.1	0.2	0.2	0.3	**0.037**

**Table 3:** Cytofluorimeter assay on HL-60 and K-562 cell lines. Percentages of cells expressing TIM3 and Dectin-1 are reported as median and interquartile range (IQR) calculated for three experiments.

Surface Molecule	Cell Line	Median (IQR)
**TIM3**	HL60	12.4 (1.7)
	K562	78.0 (5.5)
**Dectin-1**	HL60	12.1 (2.6)
	K562	75.5 (5)

## Abbreviations

APC cells	Antigen-presenting cells
APC	Allophycocyanin
Bcl-10	B-cell lymphoma/leukaemia 10
CARD9	Caspase recruitment domain 9
CD3	Cluster of differentiation 3
CD4	Cluster of differentiation 4
CD8	Cluster of differentiation 8
CLEC7A	C-Type lectin domain-containing 7A
CML	Chronic myelogenous leukaemia
COPD	Chronic obstructive pulmonary disease
DVT	Deep vein thrombosis
DVTLL	Deep vein thrombosis of lower limbs
ERK	Extracellular signal-related kinases
FITC	Fluorescein isothiocyanate
HAVCR2	Hepatitis A virus cellular receptor 2
(IL)-12	Interleukin 12
(IL)-17	Interleukin 17
INFγ	Interferon γ
MALT1	Mucosa-associated lymphoid tissue lymphoma translocation protein 1
NF-κB	Nuclear factor kappa light-chain enhancer of activated B cells
NK	Natural killer cells
PBMC	Peripheral blood mononuclear cells
PE	Phycoerythrin
SSC	Side-scattering
SYK	Spleen tyrosine kinase protein
TEM cells	Effector memory T cells
Th-1	T helper type 1
Th-17	T helper type 17
TIM	T cell immunoglobulin and mucin domain
TNF-α	Tumour necrosis factor-α
VTE	Venous thromboembolism
